# Supplementary material for: Bioaccessibility and Cellular Uptake of Carotenoids Extracted from Bactris gasipaes Fruit: Differences between Conventional and Ionic Liquid-Mediated Extraction
Source: Molecules. 2021 Jun 30;26(13):3989. doi: 10.3390/molecules26133989 (PMC8272118; doi:10.3390/molecules26133989)
Supplement: Supplementary file 1 [file molecules-26-03989-s001.zip › molecules-1241712-supplementary.pdf]

SUPPORTING INFORMATION

Bioaccessibility and Cellular Uptake of carotenoids Extracted from *Bactris gasipaes* Fruit: Differences between Conventional and Ionic Liquid-Mediated Extraction

Leonardo Mendes de Souza Mesquita [1], Daniella Carisa Murador [1], Bruna Vitória Neves[1], Anna Rafaela Cavalcante Braga[1,2], Luciana Pellegrini Pisani[1], and Veridiana Vera de Rosso [1,3*]

[1] Department of Biosciences, Federal University of São Paulo (UNIFESP), Silva Jardim Street, 136, Vila Mathias, 11015-020, Santos, SP, Brazil. mesquitalms@gmail.com
[2] Department of Exact and Earth Sciences, Federal University of São Paulo (UNIFESP), Campus Diadema, Diadema, São Paulo, 09972-270, Brazil*
[3] Nutrition and Food Service Research Center, Federal University of São Paulo (UNIFESP), Silva Jardim Street 136, Santos - São Paulo, Brazil, 11015-020. veridiana.rosso@unifesp.br

Correspondence: veridiana.rosso@unifesp.br

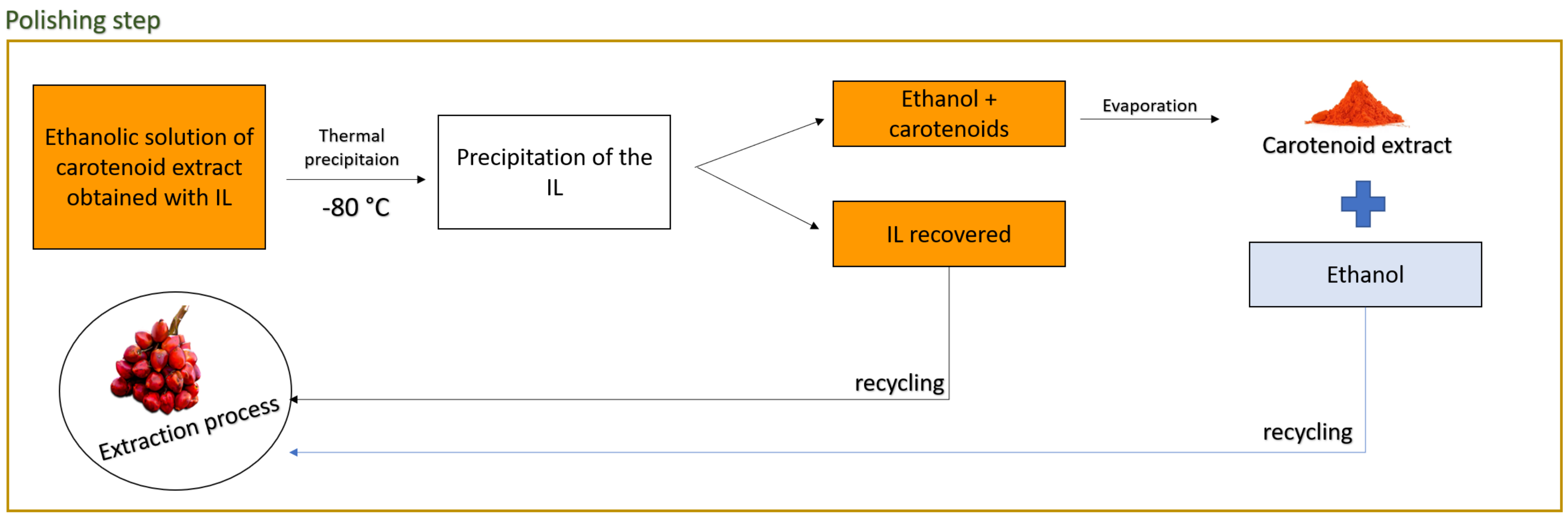

Figure S1. Representative scheme of the polishing process to separate carotenoids from the ionic liquid solvent (IL: ionic liquid).

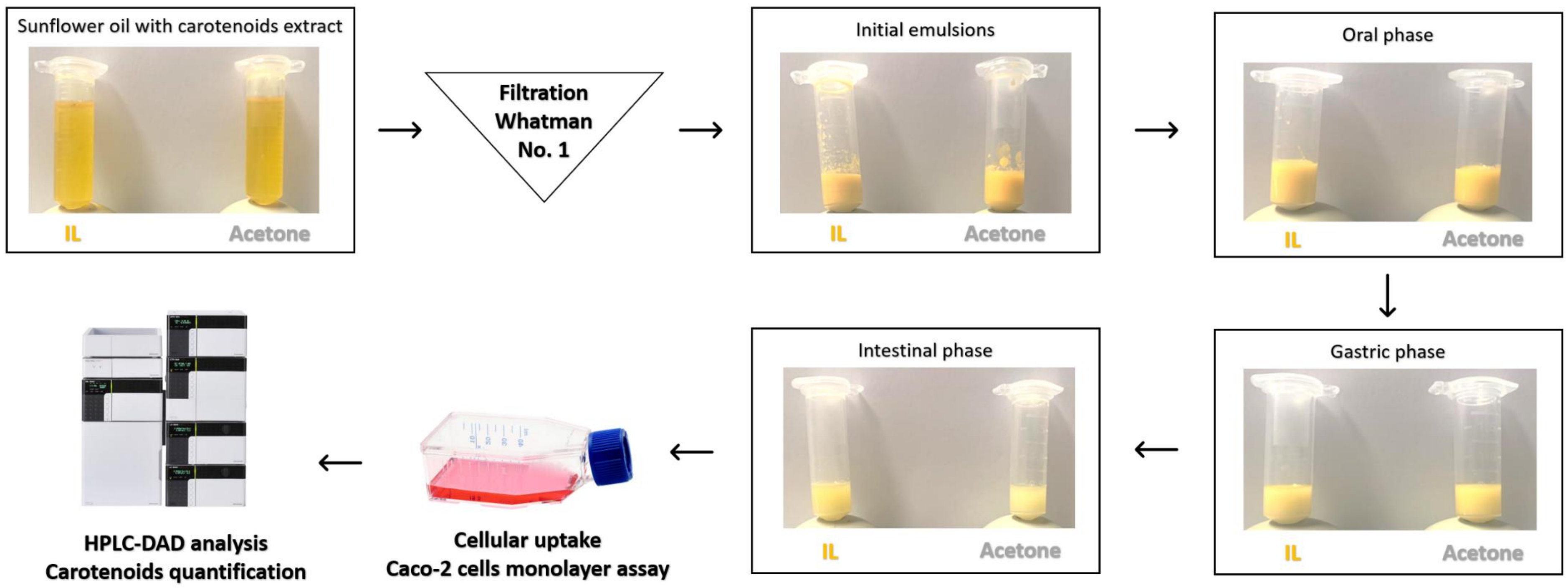

Figure S2. Representative photos representing the whole process used to compare the bioaccessibility of the extracts (IL: ionic liquid).
